# RETRACTION: Long Noncoding RNA ZNF667‐AS1 Reduces Tumor Invasion and Metastasis in Cervical Cancer by Counteracting Microrna‐93‐3p‐Dependent PEG3 Downregulation

**DOI:** 10.1002/1878-0261.13777

**Published:** 2024-11-27

## Abstract

**RETRACTION**: Y.‐J. Li, Z. Yang, Y.‐Y. Wang, and Y. Wang, “Long Noncoding RNA ZNF667‐AS1 Reduces Tumor Invasion and Metastasis in Cervical Cancer by Counteracting Microrna‐93‐3p‐Dependent PEG3 Downregulation,” *Molecular Oncology* 13, no. 11 (2019): 2375–2392, https://doi.org/10.1002/1878-0261.12565.

The above article, published online on 17 October 2019 in Wiley Online Library (wileyonlinelibrary.com), has been retracted by agreement between the journal Editor‐in‐Chief, Kevin Ryan; FEBS Press; and John Wiley & Sons Ltd. The retraction has been agreed upon following an investigation into concerns raised by a third party, which revealed implausible Western blot data (Figures 5B, L, G and Q), and an image duplication in Figure 6B. The authors' failure to respond with the original raw data has led the editors to lose confidence in the data presented. Therefore, the editors consider the conclusions substantially compromised and are retracting the paper.

**RETRACTION**: Y.‐J. Li, Z. Yang, Y.‐Y. Wang, and Y. Wang, “Long Noncoding RNA ZNF667‐AS1 Reduces Tumor Invasion and Metastasis in Cervical Cancer by Counteracting Microrna‐93‐3p‐Dependent PEG3 Downregulation,” *Molecular Oncology* 13, no. 11 (2019): 2375–2392, https://doi.org/10.1002/1878-0261.12565.

The above article, published online on 17 October 2019 in Wiley Online Library (wileyonlinelibrary.com), has been retracted by agreement between the journal Editor‐in‐Chief, Kevin Ryan; FEBS Press; and John Wiley & Sons Ltd. The retraction has been agreed upon following an investigation into concerns raised by a third party, which revealed implausible Western blot data (Figures 5B, L, G and Q), and an image duplication in Figure 6B. The authors' failure to respond with the original raw data has led the editors to lose confidence in the data presented. Therefore, the editors consider the conclusions substantially compromised and are retracting the paper.

